# Operations on the Jaws, with the Results of Thirteen Cases

**Published:** 1848-07

**Authors:** Paul F. Eve

**Affiliations:** Professor of Surgery in the Medical College of Georgia, Editor of the Southern Medical and Surgical Journal, &c.


### ARTICLE VI.

*Operations on the Jaws, with the Results of Thirteen Cases.*
By Paul F. Eve, M. D., Professor of Surgery in the Medical College of Georgia, Editor of the Southern Medical and Surgical Journal, &c.

Case 1. *Fibrous Tumor attached to Superior Maxillary Bone, with Polypus of the Nose—Ligature to both Primitive Carotids, removal of Tumor—Recovery of Patient.*

In July, 1835, I ligated the left carotid artery, removed a polypus from the left nostril, and dissected from the cheek of the same side, a fibrous tumor which was found attached to the outer surface of the left superior maxillary bone. This foreign growth had an osseous attachment, and was about the size of a guinea egg. The patient was a youth aged 18 years. His parents inhabited a sickly district of South Carolina, and had recently lost five children; this their last one was of a cachetic habit. The polypus returned the next winter, and was removed a second time. In 1836, the patient visited New York to consult Dr. Mott, who ligated the right carotid. He is now a man of family, managing a large property. I saw him three years ago, and the only evidence remaining of disease about him, was a slight fulness in the left cheek. Twelve years have now elapsed, and he has experienced no unpleasant effects from the ligature to both primitive carotid arteries.

Case 2.—*Removal of nearly the whole Right Superior Maxillary Bone for Polypus of the Antrum—Recovery of the Patient.*

May, 1836, Mr. J. S. aged 21, was operated upon the third time for a large fibrous polypus of the right antrum highmorianum. In two previous attempts the foreign growth had been attacked in the nostril, the antrum was opened, and the soft palate slit up, but without succeeding in its entire removal. In the third operation, a flap was made by two incisions through the cheek and lip, this reflected over the eye, the maxillary bone of the right side thus exposed was separated between its first and second incisor teeth, then the nasal process of the same bone divided transversely, after which the alveolar processes with six teeth (the wisdom tooth not being developed) was gradually detached. The palatine process of the superior maxilla, and the palatine plate of the palate bone were also removed, and as the fibrous tumor could not yet be pulled away, even by great force, it was separated by curved scissors from the basilar processes of the occipital and sphenoidal bones, and also from the internal plate of the pterygoid process. The mass removed weighed three ounces three and a half drachms. Three sutures were applied to the palate and five to the face in dressing the wound. The latter united, but the former did not. The patient entirely recovered: I have seen him several times since the operation, and believe he is still living in one of the upper counties of this state. (For further particulars, see Southern Medical and Surgical Journal, vol. i, first series, p. 78.)

Case 3.—*Removal of 4¾ inches of the Inferior Maxillary Bone, including one of its angles, for Osteo-Sarcoma—the disease returned and destroyed life in about* 8 *months after the Operation.*

May 31st, 1838, I operated upon a negro woman, aged 25, for a tumor involving nearly the whole left half of the lower jaw-bone. She had had one child; the catamenia were now suppressed; for years she had been complaining from what was called jaw-ache; and the molar and bicuspid teeth of the diseased side had been extracted. While preparing the patient for it, the operation was hastened from impending suffocation. An incision was made perpendicularly from the left angle of the lips to the thyroid gland, and from this point another one extended to the lobe of the ear of the same side. Extracting the canine tooth of this side, the inferior maxilla was divided with a saw, and then by careful dissection, this portion of the bone was drawn outwards so as to apply the saw at its neck, leaving its condyle in the articulation. It measured 4 inches and ¾ in length; the diseased mass presented a large fungous growth containing numerous irregularly shaped spiculæ and laminæ of bone.

Three ligatures and some eight or ten sutures with the ordinary dressings were applied, and the patient returned home on the fortieth day after the operation. She, however, did not entirely recover; the disease reappeared in the right sub-maxillary gland, then invaded all the tissues, and destroyed life in about eight months. (For further particulars, see *Southern Med. and Surg. Journal*, 1st series, vol. ii, p. 720.)

Case 4.—*Operation for Separating the Jaws—Success only Partial.*

Early in 1840, I made an attempt to re-open the mouth for the son of Mr. S., aged five years. He had had gangrænopsis, which had resulted not only in destruction of the soft parts but ankylosis of the lower jaw. There was also great deformity of the mouth. After free division of the zygomatic muscles and other soft parts, the right commissure of the lip was depressed, and the separation of the lower jaw increased by the lever power. The patient, who was from the country was committed to his parents, with instructions how to treat the case. I frequently see him, now become quite a lad, and regret to add the deformity has only partially been remedied

Case 5.—*Fungous Hæmatodes involving the Jaws—Operation—Death of the Patient in a few months after it.*

In August, 1840, I made an incision into a tumor situated under the right masseter muscle and about the size of a common egg. The patient was the Rev. Mr. D. of South Carolina, aged about 40 years. The cause of his disease was quite obscure. When the opening was made, the finger soon turned out a considerable quantity of brain-like matter, mixed with blood, and the coronoid process of the right side of the inferior maxilla down to the angle of the bone was found perfectly denuded of its periosteum. The operation was now abandoned. An immense bloody tumor was rapidly developed on the side of the face, involving all the tissues, bleeding spontaneously, and death alone relieved the sufferer in about eight months from the operation.

Case 6.—*Removal of Portion of the Inferior Maxillary Bone for Epulis—Recovery of Patient.*

On the 7th of September, 1840, I operated upon Mrs. H. in Madison, Geo., for epulis of the lower jaw. Exposing the bone by an incision from the left angle of the mouth through the cheek to the extent of an inch and a half, the diseased mass was isolated by two perpendicular applications of the saw, and it was then chipped off with the chisel and mallet. This patient fully recovered.

Case 7.—*Removal of nearly the Whole Right Superior Maxillary Bone for Fungous Hæmatodes of the Antrum, &c.—Return of the Disease and Death of the Patient in a few months.*

The 6th of February, 1842, I performed before the medical class of our college a similar operation to the one described in case 2. The patient was a negro man, aged 45 years. The disease was removed as far as could be traced, and was pronounced fungous hæmatodes or soft cancer. The patient so far recovered that he returned home in about a month, but his affection was rapidly reproduced, and he died in two or three months after the operation.

Case 8.—*Operation for Separating the Jaws—Success only Partial.*

In 1843, I operated upon Dr. G. then a student of medicine, for deformity of the mouth and closure of the inferior jaw, said to be effects of cancrum oris, or of profuse salivation. The operation and its results were similar to case 4.

Case 9.—*Trephining the Antrum Highmorianum—Death of Patient within fifty hours after the Operation.*

This case was a young lady, who for years had a tumor developing in her right cheek. Considering it the result of polypus of the antrum, the anterior surface of this cavity having been exposed, a trephine was applied and made to penetrate one inch and a half—the tumor, however, proved to be osseous. This was on the 12th of April, 1844. The operation was now abandoned; and the patient to our surprise, apparently doing well, died on the third day from symptoms of congestion, &c., of the brain. (For further particulars of this case, see *Southern Med. and Surg. Journal*, vol. iv, new series, p. 279.)

**Case 10.**—*Removal of a Portion of both the Superior Maxillary Bones for Epulis—Recovery of Patient.*

Mrs. H. of South Carolina had been laboring for some years under a fungous growth from the alveolar processes of the upper jaw. It proceeded from carious teeth, some of which had been plugged and others had artificials inserted upon their stumps or roots. The tumor now extended to both of the superior maxillary bones. The diseased mass had once been removed by excision. On the 5th of July, 1845, I divided the gums both anteriorly and posteriorly to the foramen incissivum; then by extracting the first molar of one side and the canine tooth of the other, with saw, &c., the included portion of the bone was removed. The patient had a considerable hemorrhage during the evening after the operation; having been called into the country, another physician kindly arrested it by creosote. Mrs. H. left for home a week after the operation, and had a good recovery. A small tubercle subsequently presented itself, but was promptly repressed by caustic, and I believe she is still quite well.

**Case 11.**—*Removal of a Portion of the Inferior Maxillary Bone for Osteo-Sarcoma—Recovery of Patient.*

Juno, a negro woman, aged 30 years, had carious teeth, and by the extraction of a molar tooth of the left side of the inferior maxilla, a portion of the alveolar was removed with it. She has continued to suffer pain in the jaw for some years past; and has had all the teeth of this side extracted. Eighteen months ago, a tumor began to be developed at the seat of pain, which was laid open by a physician. On the 14th of October, 1845, thinking it a case of epulis, the whole of the diseased mass was cut away, and as it began to be reproduced, I applied the actual cautery on six different occasions. The knife in removing the tumor had its edge fractured by coming in contact with spiculæ of bone which it contained. On the 16th of November same year, I divided the cheek from the left commissure of the mouth to near the angle of the lower jaw of the same side. This exposed the whole disease of the bone which when denuded was removed with the saw. The artery of the bone was found obliterated; a mere strip of its base remained undivided by the saw.

This patient had a good recovery; was presented to the class on the 23d, seven days after the operation, apparently cured; and returned home on the 25th. I saw Juno a year after the operation, and found her not only healthy and sound, but the mother of another infant.

Case 12.—*Removal of Portion of the Inferior Maxillary Bone for Osteo-Sarcoma—Recovery of Patient.*

June 5th, 1846, I operated upon Mrs. D., in Decatur, Ga., for disease of the lower jaw-bone. It had been of some years standing, and involved the whole circumference of the bone. It was about the size of a turkey egg, its center occupying the right stomach tooth, and included the base of this bone. A plug of the inferior maxilla, about one inch and a half of its whole circumference was removed and contained thin spicula and lamina of osseous structure. This patient had a good recovery, and continues well at present.

Case 13.—*Operation to Separate the Jaws—Failure.*

In 1846, Mrs. W., of South Carolina, applied to have her mouth re-opened, and a large circular opening closed in her left cheek. The deformity was attributed to profuse salivation. Slitting the left commissure of the mouth, a lever was applied upon the lower jaw, but no force exercised could start the bone at its articulation. The portion of the alveolar holding the inferior incisor teeth upon which the lever was applied was even fractured. The incision of the soft parts was then extended to the coronoid process, and the saw applied to it, but again without success in separating the jaw. A ledge of bone, or a coalescing of the coronoid process to the malar bone seemed to have been produced or to have occurred in this case. Closing the opening in the cheek, Dr. Mott's instrument for separating the jaws was furnished the patient, and after a few days she returned home. I have since learned that she has not been much benefited by the operation.

In four of the above cases the operation was limited to the superior maxillary bone, in four others to the inferior maxilla, and in the five remaining it included both of the jaw-bones. In one, death occurred within three days after the operation, and in three others the patients only partially recovered from the disease—not one dying from the effects of the operative procedure in itself.

I am now engaged with a case requiring operation upon the jaws.

